# Nanoparticles for Bio-Medical Applications

**DOI:** 10.3390/nano12071189

**Published:** 2022-04-02

**Authors:** Miguel Gisbert-Garzarán, María Vallet-Regí

**Affiliations:** 1 Institut Galien Paris-Saclay, UMR 8612, CNRS, Faculté de Pharmacie, Université Paris-Saclay, CEDEX, F-92296 Châtenay-Malabry, France; 2 Departamento de Química en Ciencias Farmacéuticas, Universidad Complutense de Madrid, Instituto de Investigación Sanitaria Hospital 12 de Octubre i + 12, Plaza Ramón y Cajal s/n, 28040 Madrid, Spain; 3 Networking Research Center on Bioengineering, Biomaterials and Nanomedicine (CIBER-BBN), 28029 Madrid, Spain

The Special Issue of *Nanomaterials* “Nanoparticles for Biomedical Applications” highlights the use of different types of nanoparticles for biomedical applications, including magnetic nanoparticles, mesoporous carbon nanoparticles, mesoporous bioactive glass nanoparticles, and mesoporous silica nanoparticles. The wide variety of applications covered by the 16 articles published here are proof of the growing attention that the use of nanoparticles has received in recent years.

Nanoparticles can find application in bone diseases. For instance, Estévez and coworkers developed biocompatible electrospun scaffolds made of type I collagen containing superparamagnetic iron oxide nanoparticles [1]. The authors found that the magnetic properties were kept after incorporating them within the matrix, paving the way for exploring the use of a magnetic stimulus for bone regeneration. Narayanaswamy et al. also explored the synthesis of magnetic nanoparticles with potential biomedical application [2]. In relation to bone diseases, Kavasi and coworkers prepared an article on the biocompatibility of nanohydroxyapatite particles [3]. These nanoparticles showed an absence of toxicity on MC3T3-E1 cells and lay the foundations for further applications in bone regeneration, tooth implants, and drug delivery. Mutlu et al. prepared SiO_2_-CaO hollow mesoporous bioactive glass nanoparticles for bone applications using a combination of etching and impregnation strategies [4]. The authors demonstrated that differentiation of MC3T3-E1 cells occurs after incubation with these nanoparticles, showing their effectiveness in bone regeneration. Similarly, Casarrubios and coworkers reported calcium-containing mesoporous nanospheres loaded with ipriflavone [5]. The results demonstrate that these nanoparticles were able to promote the expression of VEGFR2 and angiogenesis, which could help to deal with osteoporosis.

Nanoparticles have long been applied to cancer treatment. For instance, Alfei et al. prepared polystyrene-based cationic nanomaterials [6]. The authors found that their formulations could generate a high amount of reactive oxygen species, which was highly effective against etoposide-resistant neuroblastoma cells and could be further explored in combination with chemotherapeutic drugs. Candela-Noguera and coworkers designed dendrimer-like mesoporous silica nanoparticles for cancer treatment [7]. The authors were able to transfect a plasmid encoding for β-galactosidase, which was able to convert a doxorubicin prodrug into active doxorubicin once inside the cancer cells. Möller et al. reported crosslinked cyclodextrin nanoparticles [8]. The authors showed the anticancer activity of curcumin in a number of cancer cell lines, demonstrating the utility of this biocompatible nanocarrier. Forsback and coworkers reported a biodegradable silica depot containing triptorelin [9]. This biomaterial could be applied to prostate cancer treatment and outperformed commercially available Pamorelin in vivo. Nanoparticles can be used not only for therapy, but also for diagnostics. In this sense, Geetha Bai and coworkers prepared a biosensor based on reduced graphene oxide for the detection of cancer [10]. The authors decorated the nanoparticles with folic acid, being able to detect overexpressed folate receptors from cancer cells with accuracy. It is also important to determine the bioavailability of nanoparticles upon different routes of administration. In this regard, Mamai et al. evaluated the biodistribution of orally administered mesoporous carbon nanoparticles [11]. The authors showed the biocompatibility of this type of nanomaterial, demonstrating accumulation in the gastrointestinal tract and complete elimination from the organism within 24 h.

Complete characterization of nanoparticles is often needed for understanding their biological behavior. In this regard, Azor-Lafarga and coworkers reported on the usefulness of atomic resolution electron microscopy for characterizing different types of nanomaterials and understanding their biological behavior [12].

Our Special Issue also covers some high-quality review articles. For instance, Aguilera-Correa et al. provide an in-depth description of the use of inorganic and polymeric nanoparticles for the treatment and prevention of viral and bacterial infections [13]. Also in relation to infectious diseases, Gheorghe and coworkers reported on the use of nanoparticles against the particular case of inner ear infections [14]. Finally, the field of stimuli-responsive nanoparticles has also been reviewed within this Special Issue. For instance, Longo et al. described the recent advances of electromagnetically responsive biomaterials [15]. Finally, Gisbert and coworkers reported the recent updates on redox-responsive mesoporous silica nanoparticles applied to cancer treatment [16].

In summary, this Special Issue presents several examples of the latest advancements on nanoparticles for biomedical applications. We hope the readers will enjoy reading these articles and find them useful for their research.
